# Circadian disruptions and their role in the development of hypertension

**DOI:** 10.3389/fnins.2024.1433512

**Published:** 2024-08-07

**Authors:** Raymond Crowthers, Trinh Thi Mong Nguyen, Diana Martinez

**Affiliations:** Department of Biomedical Sciences, Cooper Medical School of Rowan University, Camden, NJ, United States

**Keywords:** synaptic transmission, circadian system, autonomic nervous system, hypertension, circadian misalignment, shift work, social jetlag, sleep

## Abstract

Circadian fluctuations in physiological setpoints are determined by the suprachiasmatic nucleus (SCN) which exerts control over many target structures within and beyond the hypothalamus via projections. The SCN, or central pacemaker, orchestrates synchrony between the external environment and the internal circadian mechanism. The resulting cycles in hormone levels and autonomic nervous system (ANS) activity provide precise messages to specific organs, adjusting, for example, their sensitivity to approaching hormones or metabolites. The SCN responds to both photic (light) and non-photic input. Circadian patterns are found in both heart rate and blood pressure, which are linked to daily variations in activity and autonomic nervous system activity. Variations in blood pressure are of great interest as several cardiovascular diseases such as stroke, arrhythmias, and hypertension are linked to circadian rhythm dysregulation. The disruption of normal day-night cycles, such as in shift work, social jetlag, or eating outside of normal hours leads to desynchronization of the central and peripheral clocks. This desynchronization leads to disorganization of the cellular processes that are normally driven by the interactions of the SCN and photic input. Here, we review autonomic system function and dysfunction due to regulation and interaction between different cardiorespiratory brain centers and the SCN, as well as social, lifestyle, and external factors that may impact the circadian control of blood pressure.

## Introduction

Circadian rhythm, or circadian cycle, is a natural oscillation that repeats roughly every 24 h (Jonathan Sobel, 2019). Circadian rhythms exist in body temperature (Mellette et al., 1951), hormone secretion (Retiene et al., 1966), glucose homeostasis (Van Cauter et al., 1991), sleep–wake cycles (McGeer and McGeer, 1966), and metabolism (Marcheva et al., 2010). Biological rhythms have served to help humans adapt to environmental changes, temperature, and food availability (Greenwell et al., 2019).

For several decades, it has been recognized that blood pressure follows a circadian rhythm in humans. This regularity of blood pressure is found in mouse and rat models, which are often used to simulate human cardiovascular physiology. According to the American Heart Association, a ‘normal’ blood pressure range is below 120/80 mmHg, an elevated pressure as 120–129/<80 mmHg, a stage 1 hypertensive BP as 130–139/80–89 mmHg, and a stage 2 hypertensive BP as ≥140/90 mmHg (Whelton et al., 2017). Importantly, hypertension cannot be diagnosed with a singular reading; it must be consistently elevated over multiple readings. Furthermore, the lower limit of hypertensive BP has continued to drop (2003, 140/90 mmHg, 2017, 130/80 mmHg), signifying the continuously evolving understanding of what even slightly increased BPs can amount to over time (Chobanian et al., 2003). Due to its impact on chronic cardiovascular disorders and complex events such as strokes and heart failure, blood pressure is one of the most researched physiological variables with diurnal variation.

**Figure 1:**
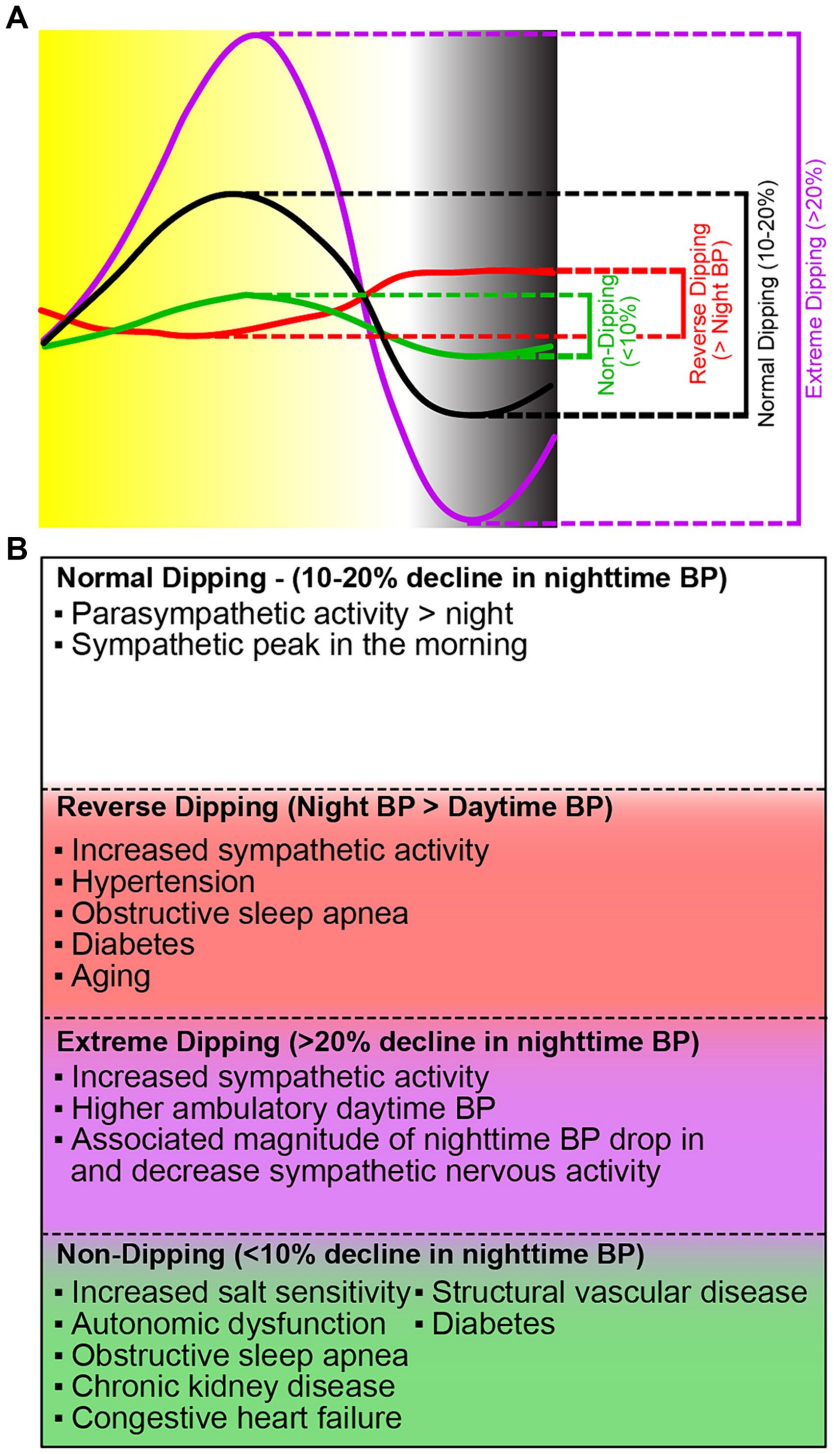
Different dipping patterns of nocturnal BP surge and their causes. **(A)** The fall in blood pressure (dip) is the difference between daytime mean systolic and daytime mean nighttime pressure. There are four categories of blood pressure dipping. Normal dipping (black) is categorized as a daytime and nighttime difference between 10 and 20%. Non-dipping (green) is a daytime and nighttime difference of <10%. Extreme dipping (purple) is a daytime and nighttime systolic blood pressure difference of greater than 20%. Reverse dipping (red) is defined as the nighttime systolic blood pressure greater than daytime systolic blood pressure. **(B)** Current knowledge on the causes of the different blood pressure dipping patterns.

Blood pressure follows a 24-h rhythm (Figure 1). Blood pressure falls (“dips”) during sleep, rises sharply in the morning (known as the “morning surge”), and normally peaks in the late afternoon (O'Brien et al., 1988). The morning surge is thought to be regulated by the sympathetic nervous system; however, the entire mechanism is unclear. Blood pressure dipping is thought to be beneficial for the cardiovascular system (Staessen et al., 2001). Among individuals who do experience blood pressure dipping, blood pressure decreases by approximately 20% between sleep and wake. Blood pressure that does not decrease by at least 10% from wake to sleep is termed “non-dipping” and is associated with an increased risk for hypertension-mediated organ damage, cardiovascular morbidity, and mortality (Lurbe et al., 2002). Non-dipping hypertension occurs in as many as 45% of individuals overall, and prevalence is even higher in patients treated with hypertensives (Sierra et al., 2009). Though there is not one specific known cause of non-dipping blood pressure, the associated correlations can generally be categorized as factors that lead to, or indicate, the dysregulation of the autonomic nervous system and/or water-salt control in the body; these include but are not limited to hyperuricemia, endothelial dysfunction, changes in sleep timings, high salt diet, belonging to the Black population, and aging (Hou et al., 2000; Kario et al., 2003; Agyemang et al., 2005; Hinderliter et al., 2013; Thomas et al., 2020; Koike et al., 2023). Furthermore, the loss or reversal of nighttime dipping is an important indicator for accelerated atherosclerosis, coronary artery disease, and stroke (Kong et al., 2020). With such an extensive list of factors (genetic components, habits, lifestyles, accumulated lifelong maladies) associated with the acquisition of non-dipping blood pressure, and thereby cardiovascular morbidity and mortality, it is important to delve further into their relationship.

While the relationship between hypertension and changes in the circadian rhythm of blood pressure is still a subject of research, it is far from settled and there are a myriad of proposed mechanisms (Millar-Craig et al., 1978; Licht et al., 2013; Douma and Gumz, 2018; Cuspidi et al., 2019; Martinez et al., 2020; Viggiano et al., 2023). A nuanced understanding of how these systems interact to maintain cardiorespiratory homeostasis throughout the 24-h day is needed to direct possible therapeutic interventions. Here, we highlight recent evidence for the role of cardiorespiratory brain regions in regulation of blood pressure and investigate social factors (e.g., shift work, sleep disruptions, diet, and activities) in the regulation of circadian blood pressure in normal and abnormal sleep conditions.

## The SCN acts as an autonomic circadian pacemaker through interactions with brain nuclei and it can be disrupted

The suprachiasmatic nucleus (SCN) of the hypothalamus encodes information about the body’s state to synchronize all internal clocks with the external environment. Based on morphological differences and neurochemical content, the SCN can be thought of as containing two different sub-regions, a ventrolateral core and a dorsomedial shell (Silver et al., 1996) that seem to modulate each other. It has been indicated that vasoactive intestinal peptide SCN core neurons could modify arginine vasopressin SCN shell neuronal expression, which in turn may change gastrin-releasing peptide expression (Shruti et al., 2018).

**Figure 2:**
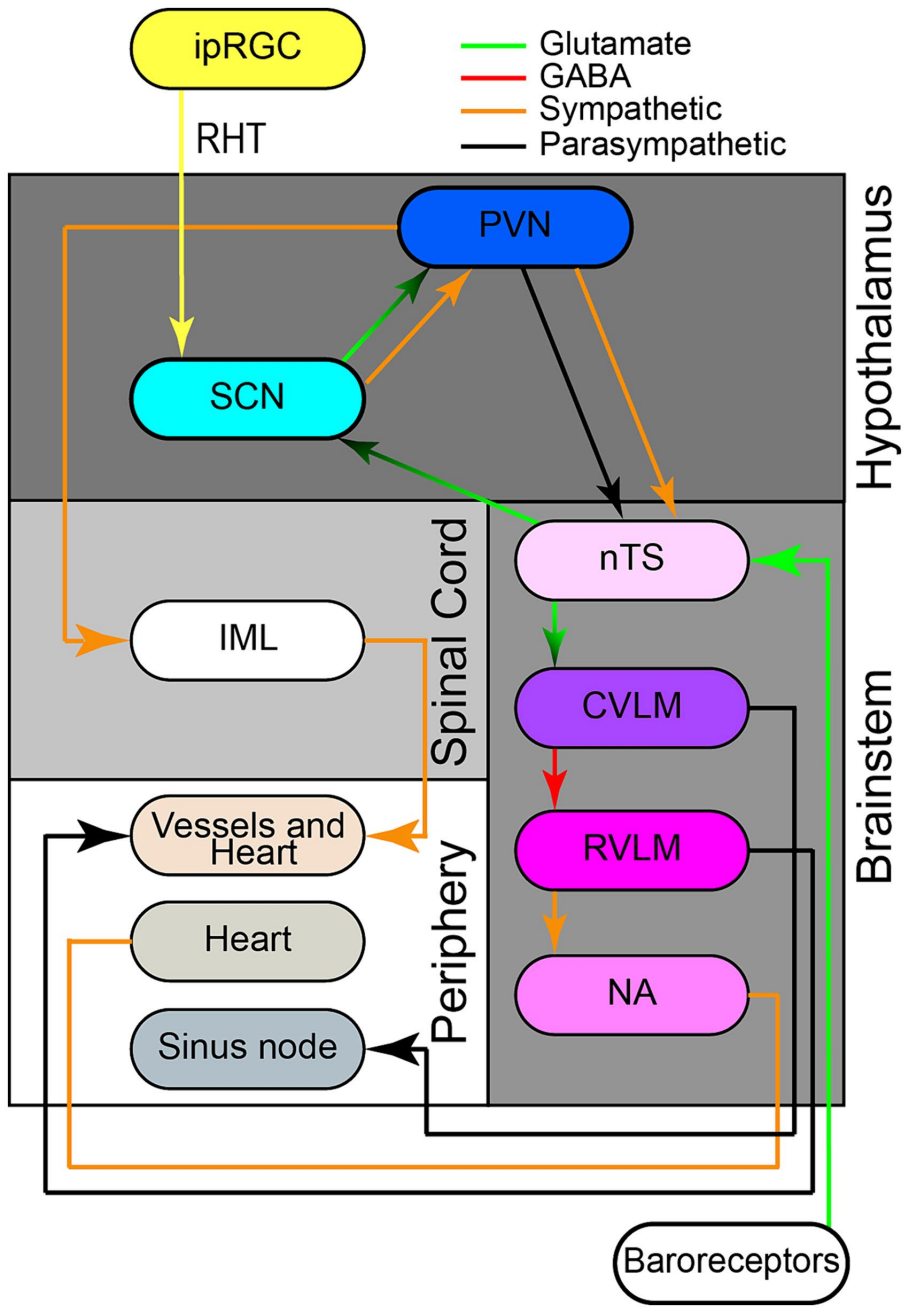
Neuroanatomical network involved in 24-h blood pressure control. The retinohypothalamic tract (RHT) originates from intrinsically photosensitive retinal ganglion cells (ipRGCs), transmitting photic information via monosynaptic glutamatergic pathways through the optic nerve and chiasma to the ventrolateral suprachiasmatic nucleus (SCN). The SCN receives glutamatergic inputs from both the RHT and the nucleus of the solitary tract (nTS). The SCN also projects glutamatergic pre-autonomic parasympathetic or parasympathetic neurons to pre-autonomic neurons paraventricular nucleus (PVN). Separate sympathetic and parasympathetic pre-autonomic neurons from the PVN project to intermediolateral nucleus (IML). Additionally, pre-autonomic sympathetic neurons in the PVN form axon collaterals to pre-autonomic parasympathetic neurons in the PVN itself and to the nTS. The nTS, located in the brainstem, receives information and signals from aortic and carotid baroreceptors, traveling via glossopharyngeal and vagal nerves respectively, to activate nTS neurons after an increase in blood pressure. The nTS then excites caudal ventrolateral medulla (CVLM) via glutamatergic fibers, which in turn inhibits rostral ventrolateral medulla (RVLM) through GABAergic projections. RVLM neurons project to sympathetic pre-ganglionic neurons in the intermediolateral nucleus (IML), regulating sympathetic vasomotor tone. The IML the sympathetic and parasympathetic branches of the autonomic nervous system have opposite effects on BP. The nucleus ambiguus (nAmb) in the medullary reticular formation, receiving fibers from nTS, sends efferent motor fibers via the vagus nerve (CN X) for cardio-inhibitory parasympathetic activity during rapid BP changes. Retinohypothalamic tract (RHT); nTS (nucleus of solitary tract); Suprachiasmatic nucleus (SCN); caudal ventrolateral medulla (CVLM); rostral ventrolateral medulla (RVLM).

There are many afferent neural pathways that branch into the SCN (Figure 2). Located within the complex neural architecture of the brainstem is a network that is responsible for the control of blood pressure over a 24-h cycle. This system includes the nucleus tractus solitarius (nTS), which is a major integration center for cardiovascular input. Blood pressure changes are monitored by baroreceptors located in the large arteries which then communicates these changes to the nTS via sensory neurons. The nTS then transmits integrated signals to the paraventricular nucleus (PVN) of the hypothalamus, which acts as the center for autonomic regulation and coordinates sympathetic and parasympathetic response (Reddy et al., 2005). This influences vascular tone, heart rate, and blood pressure. Additionally, the SCN located within the hypothalamus functions to control circadian rhythm. The SCN receives external light signals from the retinohypothalamic tract (RHT) via monosynaptic glutaminergic pathways and projects these glutaminergic inputs to the PVN (Johnson et al., 1988). Ultimately, these signals integrate the environmental day-night cycle with the human body’s internal clock. The connections between the nTS, PVN, and SCN coordinates the timing of blood pressure fluctuation with periods of wakefulness and sleep.

The SCN receives non-photic information from the body through the geniculohypothalamic (Janik and Mrosovsky, 1994) and the raphe-hypothalamic tracts (Abrahamson and Moore, 2001). However, the primary tract of the SCN is the RHT (Dark and Asdourian, 1975), which originates from photosensitive ganglion cells in the retina (Gooley et al., 2001) and project to the core of the SCN after stimulation from an environmental light source (Abrahamson and Moore, 2001; Muscat et al., 2003). Here, the neurons can cycle between day depolarization and night hyperpolarization, increasing or decreasing GABA release at their target site, respectively. In the SCN, there are acute and tonic responses to light. Together, these oscillating networks set the body’s idea of day-length (van Beurden et al., 2022), which is critical for coordinating molecular circadian rhythms in organs and cells throughout the body (Yoo et al., 2004). Interestingly, the shell and core SCN neurons are slightly out of phase with each other. The reason suggested for this slight phase difference lies in the idea of ‘anticipated’ activity. As mentioned above, the core is the efferent site of optic information. Therefore, in order to prepare, the shell activity peaks just before the core in ‘preparation’ (Goltsev et al., 2022).

Additionally, studies have shown that the release of glutamate in the nTS follows a circadian pattern, peaking during the light phase of the day as light triggers the release of glutamate initiating a signal transduction cascade in the neurons of the SCN. This leads to a phase shift in the circadian rhythm (Michel et al., 2002). Circadian regulation impacts neuronal activity by influencing the excitability of intrinsic membrane properties of the nTS neurons. During light phase, enhanced glutamate release has been shown to modulate responsiveness of nTS neurons to afferent input. These changes result in higher basal neuronal firing rates. The circadian rhythm, combined with glutaminergic activity and membrane conductance in the nTS, drives day-night variations in both basal and afferent-evoked firing (Ragozzino et al., 2023).

Four families of essential clock genes (*clock*, *bmal1*, *period*, and *cryptochrome*) produce a transcription-translation feedback loop in all nucleated cells that cycles every 24 h (Buhr and Takahashi, 2013). Positive regulators *clock* and *bmal1* dimerize and initiate transcription of the *period* and *cryptochrome* genes. Because they limit *clock::bmal1* transactivation and thus turn off their own expression, translated *period* and *cryptochrome* proteins are negative factors in the feedback loop. There are other interconnected molecular route loops, such as the rhythmic transcription of Rev-erb and Ror, which controls *bmal1* expression cycles (Guillaumond et al., 2005; Zhang et al., 2021). The feedback process involves huge protein complexes that include chromatin modifiers (Duong and Weitz, 2014; Kim et al., 2014), although this process is still poorly understood. One gene thought to contribute to the expression of glutamate excitatory amino acid transporter 2 (EAAT2) and thereby the expression of glutamate in the SCN is beta-catenin. Increased reuptake of glutamate by EAAT2s in the morning leads to a reduced level of glutamate in the synaptic cleft, decreased activation of the SCN, and can, ultimately, lead to an increase in BP during the day (Lutgen et al., 2016). This cycle of expression activation and inhibition has a hand in creating the physiological 24-h blood pressure patterning.

The SCN projects to the hypothalamus, where the SCN monosynaptically influences the hypothalamic output to the main vagal motor nucleus and the sympathetic preganglionic motor neurons of the spinal cord. Through constant release of glutamate in the PVN, the SCN signals the pineal gland to release melatonin during the dark phase, which is prevented through the diurnal rhythm of SCN-induced release of GABA into the PVN. Light activates other SCN neurons and inhibits (via GABA) the same PVN neurons (Hermes et al., 1996), resulting in an immediate halt of secretion of melatonin and lowering of blood pressure (Perreau-Lenz et al., 2003). The SCN also projects to the dorsomedial nucleus of the DMH through a primary projection to the PVN that controls autonomic nervous system activity but may indirectly impact sleep (Horst and Luiten, 1986; Deurveilher et al., 2002).

It has been demonstrated in rodent models that the SCN has an endogenous rhythm due to the continued 24-h period of pacemaker activity when placed in complete darkness (Pittendrigh and Daan, 1976). Endogenous rhythms, such as the sleep–wake cycle and hormone secretion patterns demonstrate “free-running” in constant conditions that is regulated by the internal biological clocks. The SCN circadian system is essential for modulating these rhythms and remains intact *in-vitro*, regardless of preparation time (Vanderleest et al., 2009). Furthermore, a study by Blagonravov et al. comparing hypertensive SHR and normotensive Wistar-Kyoto rats under a 24-h light deprivation and free-run rhythm, highlights the role of the sympathetic nervous system in regulating heart rate and suggests implications for cardiovascular adaptations in response to changes in light–dark cycles (Blagonravov et al., 2018). Disruptions in the SCN circadian system have been associated with a variety of physiological problems in rodents, including erasure or attenuation of daily rhythms in locomotor activity (Schwartz and Zimmerman, 1991), temperature (Warren et al., 1994), and breathing (Purnell and Buchanan, 2020). In Sprague Dawley rats, destruction of the SCN abolished circadian rhythmicity of outputs such as blood pressure and heart rate (Witte et al., 1998). Additionally, there are many feedback mechanisms that can modify the circadian rhythm of blood pressure. Though there are wide-ranging causes of each of these interferences (sleep fragmentation/stimulation during sleep, restless leg syndrome, various means of Renin-Angiotensin-Aldosterone activation, obstructive sleep apnea, and many more pathologies), a few salient categories of pathology lead to the formation of non-dipping blood pressure and its deleterious end-results: water-salt dysregulation, increased sympathetic activity, and chronic inflammation. Though these all are attributed to the acquisition of hypertension, questions remain as to the direct pathophysiology of set-point alteration into hypertensive blood pressure ranges.

## Shiftwork, sleep dysregulation, and circadian misalignment

Circadian misalignment is defined as a non-congruent endogenous circadian rhythm and environmental (light) or behavior cycle (Morris et al., 2016). Of the many factors that can contribute to circadian misalignment, shift employment has been implicated due to its tendency to cause shorter duration sleep and interrupted sleep cycles via an atypical circadian phase (Juda et al., 2013). In the past 20 years, approximately one out of every five working Americans has participated in shift work, with 29% of full-time professionals working shifts that include evenings (McMenamin, 2007; Alterman et al., 2013; U.S. Bureau of Labor Statistics, 2019). This was of great importance during the initial stages of the COVID-19 pandemic, as essential workers, such as health care workers, were working more rotating and longer shifts and those working at home were spreading their work out over the day (Li et al., 2021; Aughinbaugh and Rothstein, 2022; Djupedal et al., 2022).

Sleep deprivation and short sleep duration (<6 h) result from circadian misalignment as suggested by shift work (Herrero San Martin et al., 2020). Circadian misalignment causes shift workers’ blood pressure to rise, in part, due to changes in the dipping status of blood pressure (Morris et al., 2016). This is especially true for those exposed to chronic shift work or who are older in age. Sleep disruptions caused by even intermittent night work prevent the nocturnal dip in blood pressure (Verdecchia et al., 1994). Additionally, shift workers may exhibit lower heart rate variability (HRV), an indicator of cardiovascular health; low HRV suggests poorer cardiac health, while high HRV suggests greater cardiovascular fitness (Hulsegge et al., 2018). Though evidence of nocturnal blood pressure dipping alteration and HRV in the setting of shift work have been demonstrated, the direct association between rotating shift work, night shift work, circadian misalignment, and hypertension are not as well defined. Some studies have found an elevated risk of hypertension among shift workers, particularly rotating and night shift workers (Morikawa et al., 1999; Suwazono et al., 2008; Lieu et al., 2012). On the other hand, other studies have found no correlation between shift work and hypertension (Murata et al., 2005; Sfreddo et al., 2010).

## Aging as a factor in circadian misalignment

Sleep disorders are prevalent in the elderly, who generally report decreased total sleep time, lower sleep efficiency, frequent night awakenings, excessive tiredness during the day, and impaired sleep adaptation due to unfavorable circadian phases and sleep-wakefulness misalignment. For example, sleep disordered breathing (SDB), insufficient sleep duration, and sleep architecture problems may influence neurohormonal axes, particularly in the sympathetic nervous system, resulting in hypertension. Over 65% of Americans over the age of 60 have been diagnosed with hypertension, which may be linked to disturbances in the normal circadian rhythm. The mechanisms by which aging affects circadian control of blood pressure are not fully understood.

Studies have shown that the 24-h blood pressure profile was markedly flattened with increasing age. Non-dipping or less dipping was associated with more brain atrophy in older participants, and both were also associated with slower gait speed and a worse functional result following stroke (Hajjar et al., 2010). Animal studies have helped shed light on possible reasons for this interruption in circadian blood pressure. Nakamura et al. (2011) examined aging mice *in vivo* and discovered decreased amplitude electrical activity from the SCN, as well as decreased neuronal efferent connections from the SCN to the hypothalamic subparaventricular zone (Nakamura et al., 2011), whereas in younger mice, SCN activity was higher (Nakamura et al., 2011). Treatment of altered circadian rhythms at the SCN level may alleviate nocturnal sleep disruptions, daytime weariness, and increases in blood pressure among some older adults.

Aging adults can also begin to lose circadian adaptation to shift work schedules. They accrue more sleep loss than younger shift workers (Rosa et al., 1996), are less able to adapt to the circadian misalignment of shift work (Härmä et al., 1994), and report higher levels of sleepiness and disrupted sleep due to shift work than younger shift workers (Härmä et al., 1994; Smith and Mason, 2001; Sack et al., 2007). A substantial percentage of shift workers develop shift work sleep disorder, which is triggered by circadian misalignment resulting in insomnia and excessive sleepiness (Akerstedt et al., 2010). Those with shift work sleep disorder also experience disrupted melatonin rhythmicity (Dumont et al., 2012; Papantoniou et al., 2014), which normally fluctuates diurnally as melatonin production peaks at night and dwindles during the day (Iigo et al., 2003). This trend was thought to become a problem over time with chronic shift work. While it is true that the degree of these effects correlates with age and the number of years completing shift work, there is evidence to suggest that even solitary night shifts can affect heart rate variability for days. However, the full length of recovery required for this single event is unknown (Li et al., 2017).

Comparisons between juveniles and adults have pointed to some possible explanations for differences in sleep and autonomic regulation between the two. Using brain tissue from the Netherlands Brain Bank, Hofman and Swaab (1993) found that among young donors (6–47 years of age) who died during the day (10:00–18:00 h), the SCN contained more than twice the number of immunocytochemically labeled vasopressin neurons as those who died at night (22:00–06:00 h) (Hofman and Swaab, 1993). The vasopressin cell count peaked in the early morning (06:00–10:00 h). This significant circadian variation is absent in donors over age 50, establishing a potential biological basis for sleep difficulties in the elderly (Hofman and Swaab, 1993). These differences in neuronal expression at older ages may account for why older shift workers do not adapt well to rotating schedules and have a higher incidence of cardiovascular events. Further work by Huang et al. (2023) has demonstrated a similar set of parameters affecting older individuals which make them more prone to circadian misalignment, mainly a decreased responsiveness to the sympathetic pathway or light and a diminished release of neurotransmitters (Huang et al., 2023). Collectively, these findings point to a decreased reactivity to circadian rhythm perturbations as humans age, and therefore, a proclivity to misalignment both through action (shift work) and age-related dysregulation (decreased responsiveness).

## Social jetlag

Social jetlag is the difference in sleep timing between work/school and free days (Wittmann et al., 2006). This is a consequence of the discrepancy between the social clock and one’s individual circadian rhythm (Sűdy et al., 2019). Social jetlag, or “living against the clock” (Roenneberg et al., 2003; Wittmann et al., 2006), affects the majority of adolescents and adults (Wittmann et al., 2006). SJL can also occur when one goes to sleep and wakes at a different time than they would during a normal weekday (Forbush et al., 2017). Social jetlag is assessed subjectively using questionnaires, such as the Munich Chronotype Questionnaire (MCTQ), which compare the phase of entrainment of work/school and free days that a person experiences. In adolescents and college-aged students, social jetlag negatively impacts academic performance (Haraszti et al., 2014; Díaz-Morales and Escribano, 2015). Many factors contribute to social jetlag in adolescents, including staying out late, the use of blue-light-emitting devices near bedtime due to increased nighttime texting, social media use, and video gaming later in the night (Hena and Garmy, 2020). It is estimated that 70% of students experience at least 1 h of social jetlag, while almost half experience 2 h or more (Wittmann et al., 2006; Roenneberg et al., 2012).

Social jetlag adversely affects the circulatory system and increases the risk of cardiovascular disease under chronic conditions (Grimaldi et al., 2016; Forbush et al., 2017). Several studies have investigated the association between autonomic cardiac function of subjects with high social jetlag and those with low social jetlag. Südy used HRV, which is the measure of variation in time between heartbeats, as a marker for autonomic control (Sűdy et al., 2019). They found an association within the high-SJL group and reduced HRV on both work and free days (Sűdy et al., 2019). Additionally, those who had 2 h or more of social jetlag had a higher resting heart rate than those with 1 h or less of social jetlag. Each hour of social jetlag is associated with an 11% increase in the likelihood of cardiovascular disease. This increase in risk is independent of sleep duration and insomnia (Forbush et al., 2017). Reducing SJL potentially reduces cardiovascular disease risk (Gamboa Madeira et al., 2021), and consequently the cardiovascular disease risk increases with sustained SJL (Roenneberg et al., 2019).

Shift workers and those with social jetlag show changes in meal patterns, including consuming more food at unconventional times (Morikawa et al., 2008). As shift workers become accustomed to working later shifts, several changes in eating habits and patterns typically occur. The frequency and quantity of meals consumed during nighttime hours generally increases as the body’s circadian rhythm adjusts to being awake and active at night. Shift workers also show an increased consumption of unhealthy foods, such as those with increased saturated fats and sugar. The adjustment in eating habits may be influenced by the body’s natural response to changes in sleeping patterns and increased hunger (Gupta et al., 2019). Shift work was identified as a risk factor for obesity and increased weight gain in a meta-analysis of 28 studies (Liu et al., 2018). Eating unhealthy foods, increased weight, and obesity are all implicated in hypertension.

## Role of food in regulating circadian blood pressure

**Figure 3:**
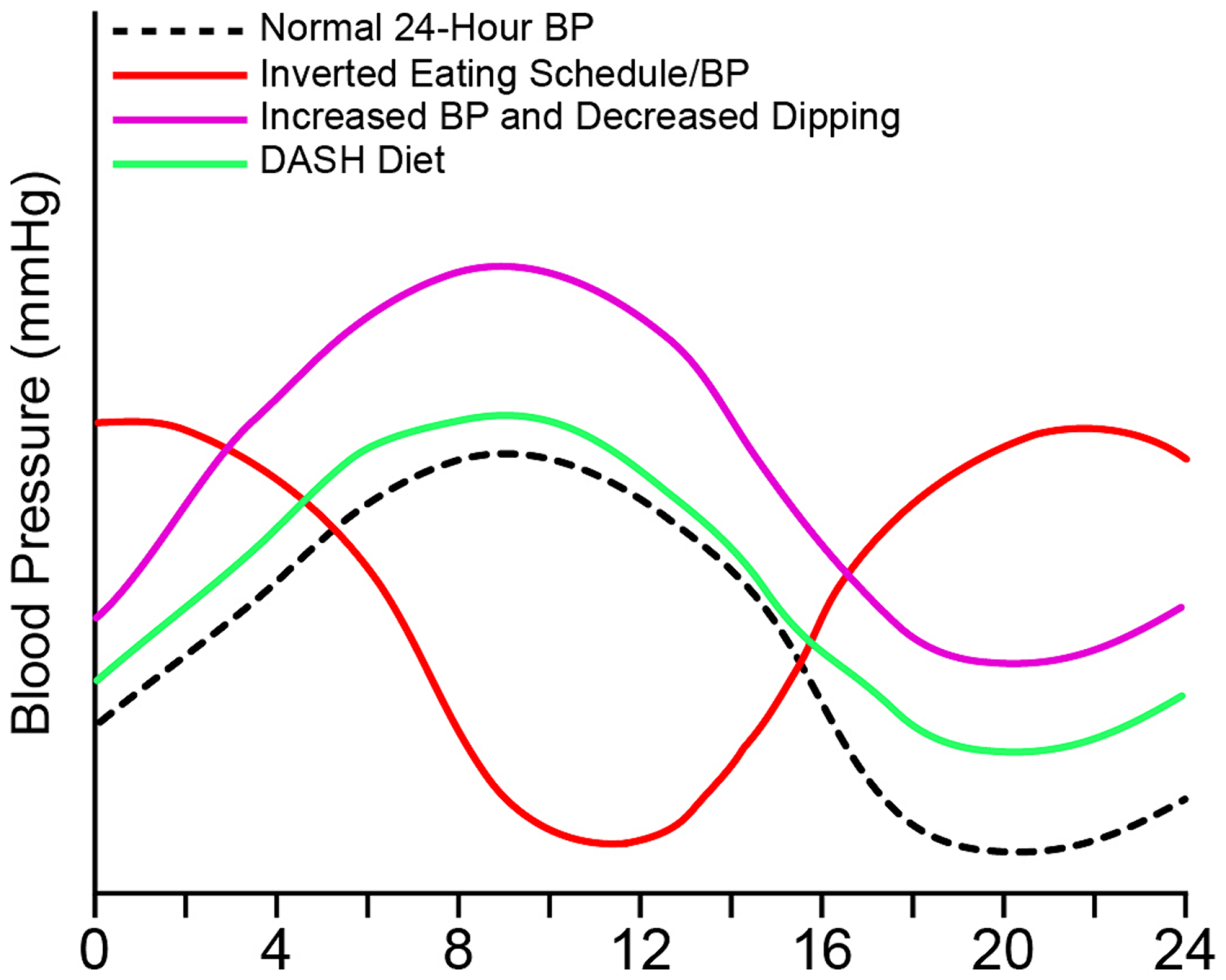
Food intake can alter 24-h blood pressure. 24-h blood pressure can be altered with timing and type of food intake. Normal blood pressure peaks in the morning and decreases at night and during sleep (black), which follows intake of food. Elevated blood pressure or hypertension can occur with an increase in weight due to increased sodium, fat, and carbohydrates (purple). Elevated blood pressure due to poor diet can be accompanied by a non-dipping pattern. The DASH diet (green) decreases blood pressure in those who are hypertensive with or without pharmacological intervention. The DASH diet is high in fruits and veggies, low in fat and sodium. An inverted blood pressure (red) occurs with overnight shifts and inverted eating schedule.

Diet can remodel the circadian rhythms of autonomic function, metabolism, and behavior. Timing of food intake has become an important factor in cardiovascular health including blood pressure (Figure 3). Zhang demonstrated the importance of the timing of food intake in the circadian rhythms of blood pressure in mice (Zhang et al., 2021). When mice were allowed food *ad libitum*, blood pressure followed diurnal rhythms—high during the active period (lights-off) and low during the inactive period (lights-on) (Zhang et al., 2021). However, when food was restricted to only the inactive period, the circadian rhythm of blood pressure was inverted. Further evidence has shown that the dorsomedial hypothalamus, when exposed to a high-fat diet, has an abatement of normal action potential generation and an over sensitization to ghrelin. These could contribute to an emergence of abnormal daytime feeding and may lend itself to the development of obesity (Palus-Chramiec et al., 2022; Sanetra et al., 2022).

In humans, weight may contribute to blood pressure changes and dipping vs. non-dipping. For instance, obesity may contribute to blood pressure abnormalities including loss of nocturnal blood pressure dipping (Pandey, 2015; Moczulska et al., 2020). A loss of 5% or more of weight was associated with an average dip increase of 8.4%, representing a significant improvement in nocturnal blood pressure. Additionally, patients gained weight increased their blood pressure by 3.2% (Pandey, 2015). In a study of 35 patients, 10 h restricted feeding reduced blood pressure and improved both cardiac and overall health (Wilkinson et al., 2020).

Restricted feeding can also affect blood pressure and its circadian rhythm. In diabetic mice, restricted feeding is associated with a non-dipping blood pressure. But imposing a diurnal food rhythm, or time-restricted feeding, prevented the loss of non-dipping blood pressure in these same diabetic mice (Hou et al., 2021). This suggests that food timing could affect the risk of hypertension among diabetics by maintaining nocturnal dipping. Studies in rodent models were so convincing that the University of Wisconsin, Madison, in collaboration with the American Heart Association, is recruiting for a clinical trial to examine correlations between meal timing and blood pressure (University of Wisconsin, Madison, 2022). The overall goal of the study is to determine whether restricted feeding normalizes blood pressure patterns and improves neurovascular control.

Growing literature demonstrates that, compared with white individuals, Black individuals are at substantially higher risk for cardiovascular morbidity and mortality (Thomas et al., 2005; Ong et al., 2007; Lloyd-Jones et al., 2010). Black individuals had higher daytime and nighttime blood pressures and an increased risk of cardiovascular disease (Yano et al., 2019). The etiology of differences in blood pressure is unclear, but dietary habits have been implicated (Langford, 1983; Douglas, 2005). One study used the Dietary Approaches to Stop Hypertension (DASH) diet, which is rich in fruits and vegetables, fiber, protein, and low-fat dairy products. The DASH diet has been found to reduce blood pressure in hypertensive patients (Appel et al., 1997). Black individuals have higher nocturnal blood pressure and a blunted dip in blood pressure (Fumo et al., 1992; Profant and Dimsdale, 1999; Jehn et al., 2008). Prather studied racial differences in nocturnal blood pressure dipping (Prather et al., 2011). The authors found that blacks randomized to regular diet, altered diet, or the DASH diet displayed marked improvement in nocturnal blood pressure dipping relative to white individuals (Prather et al., 2011).

Increased sodium intake is correlated with hypertension and loss of nocturnal blood pressure dipping. Excess sodium consumption (defined by the World Health Organization as >5 g sodium per day) (World Health Organization, 2012) elevates blood pressure significantly and has been associated with the development of hypertension and its cardiovascular consequences (Pasquale et al., 2009). Reducing sodium intake, on the other hand, not only lowers blood pressure and the incidence of hypertension, but also correlates with decreased cardiovascular morbidity and death (Kodjoe, 2022).

## Socioeconomic status, psychosocial factors, and race in blood pressure regulation

Social environment and socioeconomic status may promote non-dipping blood pressure (Stepnowsky et al., 2004). Increased cardiovascular morbidity and mortality have been observed among persons with lower socioeconomic status (Tremblay et al., 2024) who are likely to be subjected to more frequent and severe stressors, resulting in more frequent sympathetic activation. Perceived neighborhood concerns are associated with cardiovascular risk factors (Gary et al., 2008; Mujahid et al., 2008); those with more severe self-reported neighborhood concerns have less nighttime dipping in comparison to those who did not have neighborhood concerns and normal dipping (Euteneuer et al., 2014).

Race and healthcare disparities also play a role in adverse cardiovascular health, however the exact mechanism behind these discrepancies remains unclear and multifaceted (Prather et al., 2011). In non-white Hispanics, lower education and lower wealth are independently linked with more common blood pressure non-dipping in a predominantly normotensive non-white Hispanic group (Rodriguez et al., 2013). Black Hispanics are significantly more likely to be non-dippers than white Hispanic individuals (Rodriguez et al., 2013). A meta-analysis of 18 studies found that Black individuals had higher sleep blood pressure and less blood pressure dipping than whites (Profant and Dimsdale, 1999). In a large population-based clinical study, The Coronary Artery Risk Development in Young Adults (CARDIA) study, researchers investigated racial and gender differences in sleep blood pressure (Profant and Dimsdale, 1999). Black individuals had greater sleeping systolic blood pressure (SBP) and diastolic blood pressure (DBP), as well as a higher prevalence of hypertension, than white individuals. Men exhibited greater sleeping SBP and DBP and were more likely to experience nocturnal hypertension than women. Overall, sleeping blood pressure was lowest among white women, followed by white men, then Black women, and highest among Black men. In addition, SBP and DBP drop from wake to sleep was steeper in White (vs. Black) individuals, and non-dipping SBP was more prevalent in Blacks (vs. white individuals) (Profant and Dimsdale, 1999).

Many studies have examined the link between dipping and hypertension. Lead by the CDC, the Comparison of Three Combination Therapies in Lowering Blood Pressure in Black Africans (CREOLE) study was a randomized, single-blind study with uncontrolled hypertension in six countries of sub-Saharan Africa (Ojji et al., 2019). In a secondary analysis of data from this trial, the prevalence of the non-dipping blood pressure pattern was higher among Black Africans (Ingabire et al., 2021) with uncontrolled hypertension. Studies have also suggested that Mexican–Americans (Hyman et al., 2000) and Caribbean Hispanics (Phillips et al., 2000) are more likely to be non-dippers than non-Hispanic whites. In the general population, non-dipping was associated with increased total mortality and cardiovascular event (Hermida et al., 2013).

Data from the United States Bureau of Labor Statistics showed that the prevalence of shift work was highest among African Americans (23.2%) and continued to increase in their latest report published in 2019 (24.1%) [U.S. Bureau of Labor 61; 62]. Shift workers were more likely to be men and those of African American, Hispanic, or American Indian descent (Ferguson et al., 2023). Furthermore, during the COVID-19 pandemic, those most likely to be considered essential workers were also African American (Rogers et al., 2020). These individuals not only found themselves more prone to infection with COVID-19 but were also required to continue these variable working hours. In the general population, non-dipping was associated with increased total mortality and cardiovascular events (Hansen et al., 2011). Research has shown that African Americans are more likely to be non-dippers and morning surge in blood pressure being named the more important prognosticating feature of development of MACE in those with hypertension. This phenomenon of non-dipping prevalence in non-white individuals goes beyond those of African descent (Ojji et al., 2019) and extends to Mexican–Americans (Hyman et al., 2000) and Caribbean Hispanics (Phillips et al., 2000).

## Daylight savings time controversy and increase in cardiovascular incidents

In the United States, except for Arizona and Hawaii, clocks are turned forward by 1 h in the spring and backward by 1 h in the fall. Studies have shown that spring DST shift causes an elevated degree of acute myocardial infarction (Rodríguez-Cortés et al., 2023), cardiac arrest (Hook et al., 2021), and stroke (Sipilä et al., 2016). Other studies have found a 24 percent increase in the number of heart attacks occurring the Monday after initiation of DST (Amneet et al., 2014). Interestingly, stroke, myocardial infarction, and sudden cardiac death have historically had a daily pattern, occurring mostly in the morning (Muller et al., 1985, 1987).

These forward and backward shifts in time are similar to experiencing eastern and western jetlag, respectively. The SCN is able to respond to these deviations from the entrained circadian rhythm by enacting phase delays in western travel and phase advances in eastern travel. As described above, this man-made phenomenon seems to come with its consequences. A mouse model by Kilgallen et al. suggested a reason for why myocardial infarctions are more severe in the morning. They found that intact clock gene oscillators experienced hyperacute inflammation immediately preceding the infarction (Kilgallen et al., 2022). Thus, interruptions to the circadian clock, such as shifts into and out of daylight savings time (DST), may contribute to an increased cardiovascular risk.

There is much scientific, public, and political debate about DST and its presumed health consequences. The European Biological Rhythms Society (EBRS), European Sleep Research Society (ESRS), Society for Research on Biological Rhythms (SRBR), and the National Sleep Foundation declared that permanent standard time is the best option for public health. In 2019, Roenneberg et al., published a position paper about the need to abandon the idea of DST and return to Standard Time, a time when the social clock and the sun clock time are the closest match (Roenneberg et al., 2019). The recent passing of the US Senate bill to make daylight saving time permanent has been quite controversial: permanent DST would result in more light in the evening, but more darkness in the morning; this could be problematic to the circadian rhythm. The effect of this change would be felt most strongly in the winter, as daylight in many areas will not occur until close to 9 am. There is broad scientific consensus against permanent DST and in support of permanent Standard Time due to the health consequences. However, there are still some who suggest that, while the data could be correct, it is in fact rather weak, or possibly sample biased, evidence (Martín-Olalla and Mira, 2023).

## Conclusion

Circadian blood pressure is regulated by many different brain processes. These regulatory processes can be disrupted through interruptions, disruptions, or misalignment of sleep. Recent evidence demonstrates that a change in feeding time influences circadian blood pressure control and may influence the circadian misalignment faced by shift workers. While we have only presented a part of the system that regulates blood pressure, the periphery also has its own clock that works in tandem with the central clock. The SCN and other brain regions responsible for autonomic processes are likely affected by social jetlag and shift work, but the mechanism for this relationship and its long-term effects, if any, remain poorly understood. In addition to the regulatory processes presented, other mechanisms of circadian control of blood pressure should be explored in future studies. The next question to address is how cardiorespiratory regulatory brain centers are involved in circadian control of blood pressure? Future research is needed to investigate the influence of the circadian clock on downstream pathways involved in hypertension due to loss of circadian blood pressure control, which could lead to advances in our knowledge and treatment of this silent killer.

## Author contributions

RC: Writing – review & editing. TT: Writing – review & editing. DM: Conceptualization, Funding acquisition, Project administration, Resources, Supervision, Visualization, Writing – original draft, Writing – review & editing.
